# A Mendelian randomization study of genetic predisposition to autoimmune diseases and COVID-19

**DOI:** 10.1038/s41598-022-22711-1

**Published:** 2022-10-21

**Authors:** Shun Li, Shuai Yuan, C. M. Schooling, Susanna C. Larsson

**Affiliations:** 1 Clinical Epidemiology and EBM Unit, Beijing Friendship Hospital, Capital Medical University, Beijing Clinical Research Institute, Beijing, China; 2 National Clinical Research Center for Digestive Diseases, Beijing, China; 3 School of Public Health, Li Ka Shing Faculty of Medicine, The University of Hong Kong, 7 Sassoon Rd, Hong Kong, China; 4 Unit of Cardiovascular and Nutritional Epidemiology, Institute of Environmental Medicine, Karolinska Institutet, Nobelsväg 13, 17177 Stockholm, Sweden; 5 School of Public Health and Health Policy, The City University of New York, 55 W 125 St, New York, NY 10027 USA; 6 Department of Surgical Sciences, Uppsala University, Dag Hammarskjölds Väg 14B, Uppsala, Sweden

**Keywords:** Genetic association study, Viral infection, Autoimmune diseases

## Abstract

Autoimmune diseases and coronavirus disease 2019 (COVID-19) share many similarities. Concerns have arisen that autoimmune diseases may increase the susceptibility and severity of COVID-19. We used Mendelian randomization to investigate whether liability to autoimmune diseases is related to COVID-19 susceptibility and severity. Genetic instruments for 8 autoimmune diseases, including type 1 diabetes mellitus, rheumatoid arthritis, systemic lupus erythematosus, psoriasis, multiple sclerosis, primary sclerosing cholangitis, primary biliary cirrhosis and juvenile idiopathic arthritis, were obtained from published genome-wide association studies. Two-sample Mendelian randomization analyses of the associations of liability to each autoimmune disease with COVID-19 infection, hospitalized COVID-19, and very severe COVID-19 were performed using the latest publicly available genome-wide association study for COVID-19. Genetic liability to each of the autoimmune diseases was largely not associated with COVID-19 infection, hospitalized COVID-19, or very severe COVID-19 after accounting for multiple comparison. Sensitivity analysis excluding genetic variants in the human leukocyte antigen gene, which has an important role in the immune response, showed similar results. The autoimmune diseases examined were largely not genetically associated with the susceptibility or severity of COVID-19. Further investigations are warranted.

## Introduction

Similarities in clinical manifestations and immune response are evident for autoimmune diseases and coronavirus disease 2019 (COVID-19)^1^ suggesting a close relation between these diseases. Furthermore, medications for treatment of autoimmune diseases have been used in severe cases of COVID-19^2^. High type I interferon in systemic lupus erythematosus patients may involve a hyperinflammatory response in COVID-19, suggesting an increase in COVID-19 severity by inducing a hyperinflammatory response^3^. Some studies have reported higher risk of COVID-19 infection and severity of disease among people with pre-existing autoimmune conditions, such as rheumatoid arthritis (RA)^4^, while other studies have not^5,6^. A meta-analysis based on case–control studies found that the risk of COVID-19 infection was higher among autoimmune disease patients than controls, and also found a positive association of glucocorticoid use with the risk of COVID-19^7^. However, observational studies are vulnerable to confounding from other factors, such as immunosuppressant treatment for autoimmune diseases. Whether autoimmune diseases causally increase the susceptibility and severity of COVID-19 remains unknown, which may involve the priority for COVID-19 prevention and management strategies.

Here, we conducted a two-sample Mendelian randomization (MR) study to assess the associations of liability to each major autoimmune disease with susceptibility and severity of COVID-19. Ethical approval is not applicable given the summary statistics used are all publicly available.

## Results

### Selection of genetic instruments for autoimmune diseases

We obtained strong (p-values < 5 × 10^–8^) and independent (r^2^ < 0.01) genetic instruments for 8 autoimmune diseases after excluding palindromic SNPs (Supplementary Table S1). The median F-statistic for the exposures ranged from 37.9 to 56.6. The genetic variants explained 18.8% to 43.5% of the variances of the exposures.

### MR effect of each autoimmune disease on COVID-19 susceptibility and severity

**Figure 1:**
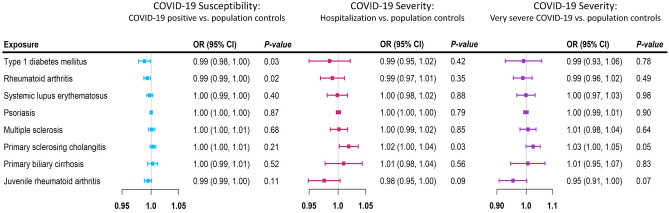
Mendelian randomization (MR) estimates for each autoimmune diseases and COVID-19. Forest plot of MR estimates of 8 autoimmune diseases with COVID-19 infection, hospitalized COVID-19 and very severe COVID-19 using the multiplicative random-effects inverse variance weighting method. The black color represents COVID-19 infection, the blue color represents hospitalized COVID-19 and the red color represents very severe COVID-19. MR estimates are reported as odds ratios per unit of log-odds of each autoimmune disease examined.

Genetic liability to each autoimmune disease was largely not associated with COVID-19 infection, hospitalized COVID-19, or very severe COVID-19 in the analyses using the IVW method (Fig. 1). The estimates were generally similar using MR-Egger and the weighted median methods (Supplementary Tables S2–S4).

Type 1 diabetes mellitus and rheumatoid arthritis were negatively associated with COVID-19 infection in the general population (Fig. 1; *p*-values for IVW = 0.031, 0.019, respectively), but were not related to hospitalized or very severe COVID-19. Primary sclerosing cholangitis, however, was positively associated with COVID-19 infection in the MR-Egger analysis (Supplementary Table S2; *p* = 0.009; *p* for intercept = 0.024) and was positively associated with hospitalized COVID-19 and very severe COVID-19 (Fig. 1; *p-*values for IVW = 0.010, 0.026, respectively). Juvenile rheumatoid arthritis was negatively associated with hospitalized COVID-19 and very severe COVID-19 in the weighted median (Supplementary Tables S3–S4; *p*-values = 0.007, 0.0003, respectively). However, none of the associations were significant after accounting for multiple comparisons in the main analysis.

**Figure 2:**
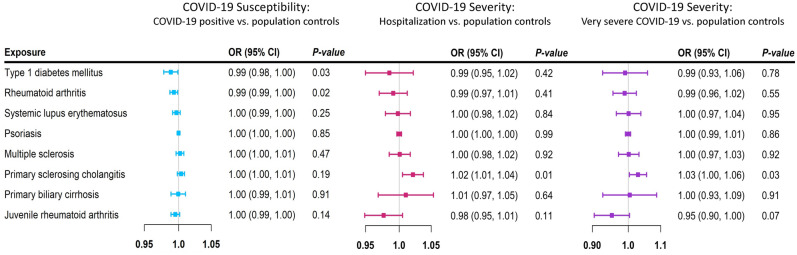
Mendelian randomization (MR) estimates of sensitivity analysis for each autoimmune disease and COVID-19. Forest plot of Mendelian randomization (MR) estimates of 8 autoimmune diseases with COVID-19 infection, hospitalized COVID-19 and very severe COVID-19 using the multiplicative random-effects inverse variance weighting method after excluding genetic variants in the human leukocyte antigen region. The black color represents COVID-19 infection, the blue color represents hospitalized COVID-19 and the red color represents very severe COVID-19. MR estimates are reported as odds ratios per unit of log-odds of each autoimmune disease examined.

Sensitivity analysis was performed by excluding the SNPs in the *HLA* gene region. The results were largely similar to those of the primary analyses (Fig. 2; Supplementary Tables S5–S7).

## Discussion

Our study provides genetic evidence with minimally confounded estimates that the genetic predisposition to autoimmune diseases examined are largely not genetically associated with the susceptibility or severity of COVID-19, which is consistent with previous findings in patients with autoimmune disorders^8^.

Potential confounders and selection bias could be major concerns in previous observational studies. A cohort study showed a positive association of RA with COVID-19 infection and hospitalization^4^. However, body mass index (BMI), which is a risk factor for both RA^4^ and COVID-19^9^ and thus a potential confounder for the association, was not adjusted for in that study. Many of the relevant studies were selected from people in middle or older age on lifelong immunosuppressant treatment^4,5,7^. Age is significantly associated with higher COVID-19 mortality and severity^10^, while autoimmune diseases affect life expectancy^11^. As such, selection of people who survived genetic liability to or autoimmune diseases and competing risk of COVID-19 may generate selection bias for examining the relation between them^12^. Another source of selection bias is from participants taking long-term immunosuppressant treatment for autoimmune diseases who were generally vulnerable to COVID-19, masking the association of autoimmune disease and COVID-19^7^. However, previous findings suggest that autoimmunity per se does not increase the susceptibility to COVID-19, independent of immunosuppressive treatment^8^.

The negative association of type 1 diabetes mellitus with COVID-19 susceptibility observed in our study was consistent with a previous MR study^9^, where significance changed due to the use of the latest GWAS for COVID-19. However, after excluding SNPs that were negatively associated with BMI, we did not find a significant association of type 1 diabetes mellitus with COVID-19 infection. An MR study showed a positive association of RA with interleukin-6 receptor (IL6R) antagonist^13^. Another MR study further showed a protective effect of IL6R inhibition on COVID-19 infection, but not on severe COVID-19, where IL6R may increase the risk of pneumonia^14^. Findings from these two studies suggest a slight positive association of RA with COVID-19 infection. However, the small effects may not be clinically significant as shown in our study.

Primary sclerosing cholangitis might be associated with higher risk of COVID-19. However, most primary sclerosing cholangitis patients after liver transplantation require lifelong immunosuppressant treatment, where liver transplantation is the only known cure for advanced disease^15^, making them vulnerable to infectious diseases^16^. A high mortality rate^17^ may also contribute to potential selection bias because the GWAS was selected on the survival^18^. Further investigation is warranted.

Some autoimmune antibodies were found in the COVID-19 patients, such as antiphospholipid antibodies^19^, while some studies have reported an increased risk of autoimmune diseases, such as type 1 diabetes mellitus after COVID-19 infection^20^. However, many of the GWAS available for autoimmune diseases were much less densely genotyped than the COVID-19 GWAS, making the reverse MR analysis less reliable. Moreover, given the auto-immune GWAS were conducted before the COVID-19 pandemic, MR studies only give pleiotropic effects of auto-immune diseases on COVID-19. Whether COVID-19 triggers the onset of autoimmune diseases requires further investigation.

Despite these findings, this study has some limitations. First, some autoimmune diseases were not included due to lack of relevant GWAS, such as Guillain–Barre syndrome. Second, we cannot fully test the relevance and exclusion restriction assumptions. However, most SNPs used were not related to potential confounders, such as BMI, and did not affect the outcome via other pathways, such as interleukin-6. Sensitivity analysis excluding SNPs in the *HLA* gene also showed consistent results. Third, potential selection bias may arise from selection on participants with a certain autoimmune disease that needs long-term immunosuppressive treatment and has a low survival rate^17^. In addition, people with autoimmune diseases may have taken more precautions to avoid COVID-19 infection than other people, which could bias towards the null. However, liability to autoimmune disease does not equate to overt disease, which may reduce this bias. MR studies are also open to selection bias, particularly for exposures that affect survival, where the sample is inevitably selected on surviving the exposure or its genetic predictors and the outcome or a competing risk of the outcome. As such a substantial proportion of deaths before recruitment from the relevant auto-immune disease could bias towards the null or even reverse the estimate. Fourth, sex-specific GWAS for autoimmune diseases and COVID-19 were not available, where sex-specific examination could be helpful as women tend to have higher risk of autoimmune diseases^21^ while men have higher risks of COVID-19 infection and severity^22^. Fifth, by examining the genetic predisposition to autoimmune diseases with COVID-19 susceptibility and severity, the study provides genetic evidence rather than the direct causal association of autoimmune diseases with COVID-19. Sixth, limited by data availability, the GWAS used mainly concern people of European descent. Caution should be taken when applying these findings to other populations.

## Methods

### Study design

This is a two-sample MR study design based on instrumental variable analysis using genetic instruments, here single-nucleotide polymorphisms (SNPs), to predict each selected autoimmune disease to test the causal relation with COVID-19. MR provides a less confounded estimate by taking advantage of the random allocation of genetic variants at conception, and is less susceptible to reverse causation^23^, but remains susceptible to selection bias^24^. MR relies on three assumptions, i.e., the genetic instruments are associated with the exposure (relevance) but are not associated with any confounder of exposure on outcome (independence) and can affect the outcome only via the exposure (exclusion-restriction)^25^.

### Data sources

#### Genetic associations with autoimmune diseases and genetic instrument selection

We included 8 autoimmune diseases as exposures, i.e., type 1 diabetes mellitus^26^, rheumatoid arthritis (RA)^27^, systemic lupus erythematosus^28^, psoriasis^29^, multiple sclerosis^30^, primary sclerosing cholangitis^18^, primary biliary cirrhosis^31^ and juvenile idiopathic arthritis (JIA)^32^. We used the largest relevant publicly available genome-wide association study (GWAS) to obtain genetic variants that were strongly (p < 5 × 10^–8^) and independently (r^2^ < 0.01) associated with each autoimmune disease^18,26,28–31^, except for RA and JIA, where the genetic variants for RA^27^ and JIA^32^ were obtained from previous well-established studies. The GWAS used were largely based on European descent individuals. Details of the GWASs, such as the number of cases and sample size, are listed in the Supplementary Table S1.

#### Genetic associations with COVID-19

Summary statistics data for COVID-19 were obtained from the COVID-19 Host Genetics Initiative GWAS meta-analysis round six (updated June 2021; without the 23andMe study), which included 112,612 COVID-19 cases *versus* 2,474,079 population controls, 24,274 hospitalized COVID-19 cases *versus* 2,061,529 population, and 8,779 very severe respiratory confirmed COVID-19 cases *versus* 1,001,875 population controls adjusted for age, sex, and ancestry^33^. A COVID-19 case was defined by laboratory based on RNA and/or serology, by physician, or by self-report.

### Statistical analysis

An approximated F statistic was used to assess instrument strength, where the square of beta for the SNP-exposure association is divided by its variance^34^.

We used inverse variance weighting (IVW) with multiplicative random effects^35^ as the main analysis. We used MR-Egger and the weighted median methods as sensitivity analysis, where MR-Egger assumes the instrument strength is independent of the direct effect (InSIDE)^36^, and the weighted median provides valid estimates when more than 50% of the information comes from valid instruments^37^. To avoid potential effects of the human leukocyte antigen (HLA), we also performed a sensitivity analysis by excluding the SNPs in the *HLA* gene given the important role of the HLA for both infectious diseases and autoimmune disorders^38^.

Analyses were conducted in R (version 4.0.3; R Foundation for Statistical Computing, Vienna, Austria). The TwoSampleMR R package (version 0.5.5) was used to extract genetic predictors and the MendelianRandomization R package (version 0.4.2) to obtain MR estimates based on the IVW, MR-Egger and weighted median methods. Bonferroni correction was used to counteract the issue of multiple comparisons (p = 0.05/8 = 0.006).

## Conclusion

Genetic liability to the autoimmune diseases examined was largely not related to susceptibility to or severity of COVID-19. Further investigations of the relation of immunosuppressive treatments and other autoimmune diseases with susceptibility to and severity of COVID-19 is warranted. Whether COVID-19 infection induces autoimmune disease requires further studies.

## Supplementary Information

Supplementary Tables.

## Data Availability

The exposure datasets generated during the current study are available in the MR-base repository, [https://gwas.mrcieu.ac.uk/datasets/], where the MR-base ID of each exposure were listed in the Supplementary Table S1. Specifically, “ebi-a-GCST005536” for type 1 diabetes mellitus, “ebi-a-GCST003156” for systemic lupus erythematosus, “ebi-a-GCST005527” for psoriasis, “ieu-b-18” for “multiple sclerosis”, “ieu-a-1112” for “primary sclerosing cholangitis”, “ebi-a-GCST005581” for primary biliary cirrhosis. The outcome datasets generated during the current study are available in the COVID19-hg GWAS, [https://www.covid19hg.org/results/r6/], where we used “COVID19_HGI_C2_ALL_leave_23andme_20210607.b37.txt” for COVID-19 infection in general population, “COVID19_HGI_B2_ALL_leave_23andme_20210607.b37.txt” for hospitalized COVID-19 and “COVID19_HGI_A2_ALL_leave_23andme_20210607.b37.txt” for very severe COVID-19. The datasets analyzed during the current study are available in the *Autoimmune disease and COVID-19* repository, [https://osf.io/sxjyc/].

## Abbreviations

BMI: Body mass index
COVID-19: Coronavirus disease 19
GWAS: Genome-wide association study
HLA: Human leukocyte antigen
IL6R: Interleukin-6 receptor
IVW: Inverse variance weighting
JIA: Juvenile idiopathic arthritis
MR: Mendelian randomization
RA: Rheumatoid arthritis
SNP: Single-nucleotide polymorphisms
